# 1-Benz­yloxy-4-(2-nitro­ethen­yl)benzene

**DOI:** 10.1107/S1600536810042960

**Published:** 2010-10-30

**Authors:** Alan R. Kennedy, Zaccheus R. Kipkorir, Claire I. Muhanji, Maurice O. Okoth

**Affiliations:** a Department of Pure & Applied Chemistry, University of Strathclyde, 295 Cathedral Street, Glasgow G1 1XL, Scotland; b Department of Chemistry and Biochemistry, Moi University, PO Box 1125-30100, Eldoret, Kenya

## Abstract

The title compound, C_15_H_13_NO_3_, crystallizes with three independent mol­ecules per asymmetric unit (*Z*′ = 3). One of these mol­ecules is found to have a configuration with a greater twist between its two aromatic rings than the other two [compare 70.26 (13) and 72.31 (12)° with 84.22 (12)°]. There are also differences in the number and nature of the weak inter­molecular C—H⋯O contacts formed by each of the three mol­ecules.

## Related literature

For discussion of C—H⋯O contacts in related derivatives, see: Gerkin (1999 ▶). For other related structures, see: Gao *et al.* (2008 ▶); Stomberg & Lundquist (1994 ▶); Wang *et al.* (2007 ▶); Zheng *et al.* (2008 ▶); Kennedy *et al.* (2010 ▶). On the design of new small mol­ecules that target HIV-1 binding sites, see: Younis *et al.* (2010 ▶); Hunter *et al.* (2008 ▶); Jones *et al.* (2006 ▶). For background to the anti­retroviral treatment programme for AIDS, see: UNAIDS/WHO (2009 ▶).
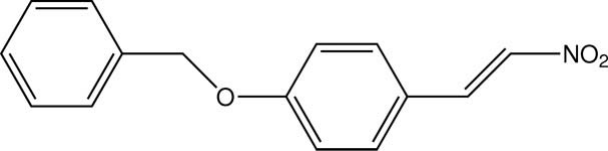


## Experimental

### 

#### Crystal data

C_15_H_13_NO_3_
*M* *_r_* = 255.26
Triclinic, 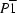

*a* = 9.9522 (8) Å
*b* = 14.0456 (13) Å
*c* = 14.2506 (10) Å
α = 74.416 (5)°
β = 84.188 (5)°
γ = 80.888 (4)°
*V* = 1891.0 (3) Å^3^
*Z* = 6
Mo *K*α radiation
μ = 0.09 mm^−1^
*T* = 120 K
0.12 × 0.10 × 0.05 mm

#### Data collection

Bruker–Nonius APEXII CCD diffractometer
25754 measured reflections
7366 independent reflections
4735 reflections with *I* > 2σ(*I*)
*R*_int_ = 0.063

#### Refinement

*R*[*F* ^2^ > 2σ(*F* ^2^)] = 0.088
*wR*(*F* ^2^) = 0.170
*S* = 1.14
7366 reflections
514 parameters
H-atom parameters constrained
Δρ_max_ = 0.27 e Å^−3^
Δρ_min_ = −0.28 e Å^−3^

### 

Data collection: *COLLECT* (Hooft, 1998 ▶); cell refinement: *DENZO* (Otwinowski & Minor, 1997 ▶) and *COLLECT*; data reduction: *DENZO* and *COLLECT*; program(s) used to solve structure: *SHELXS97* (Sheldrick, 2008 ▶); program(s) used to refine structure: *SHELXL97* (Sheldrick, 2008 ▶); molecular graphics: *ORTEP-3* (Farrugia, 1997 ▶); software used to prepare material for publication: *SHELXL97*.

## Supplementary Material

Crystal structure: contains datablocks global, I. DOI: 10.1107/S1600536810042960/si2304sup1.cif

Structure factors: contains datablocks I. DOI: 10.1107/S1600536810042960/si2304Isup2.hkl

Additional supplementary materials:  crystallographic information; 3D view; checkCIF report

## Figures and Tables

**Table 1:** Hydrogen-bond geometry (Å, °)

*D*—H⋯*A*	*D*—H	H⋯*A*	*D*⋯*A*	*D*—H⋯*A*
C3—H3⋯O8^i^	0.95	2.56	3.503 (5)	175
C8—H8⋯O8^i^	0.95	2.38	3.322 (5)	175
C5—H5⋯O9^ii^	0.95	2.58	3.493 (4)	162
C12—H12⋯O4^iii^	0.95	2.46	3.284 (5)	145
C20—H20⋯O5^iv^	0.95	2.49	3.373 (4)	155
C22—H22⋯O5^iv^	0.95	2.59	3.448 (4)	150
C18—H18⋯O6^v^	0.95	2.40	3.326 (4)	166
C33—H33⋯O3^ii^	0.95	2.40	3.325 (4)	163
C45—H45⋯O1^vi^	0.95	2.58	3.413 (5)	147

Symmetry codes: (i) 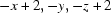; (ii) 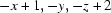; (iii) 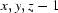; (iv) 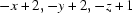; (v) 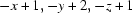; (vi) 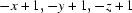.

**Fig. 1.**
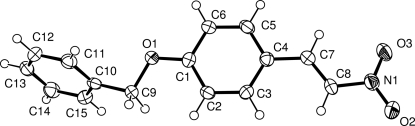
The molecular structure of one configuration of 2-(4-benzyloxyphenyl)-1-nitroethene showing 50% probability displacement ellipsoids.

**Fig. 2.**
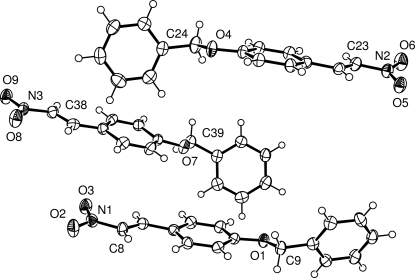
Contents of the asymmetric unit.

## supplementary crystallographic information

### Comment

One of the most serious threats to human health today is acquired immunodeficiency syndrome, AIDS (UNAIDS/WHO, 2009). Most drugs used in AIDS treatment target the HIV-1 reverse transcriptase (RT). A class of compounds known as non-nucleoside reverse transcriptase inhibitors (NNRTIs) inhibits the ability of RT to transcribe by altering its structural and dynamic properties (Jones *et al.*, 2006). In our research, (Hunter *et al.*, 2008; Younis *et al.*, 2010) *N*-[4-(2-prop-2-ynyloxyphenyl)ethyl]-*N*'-[2-(5-bromopyridyl)]thiourea, a phenylethylthiazolylthiourea analogue was the target NNRTI compound, using 4-hydroxy benzaldehyde as the starting material. In this paper, the crystal structure of a synthon of the target compound, namely 2-(4-benzyloxyphenyl)-1-nitroethene, is described as determined by *x*-ray diffraction.

The sample was recrystallized several times, and data collected from numerous crystals. Non-single-crystal samples were common and data quality tended to be low, however all data collections were consistant with that of the best quality structure found, as described below. The title compound was found to exist as discrete molecules (Figure 1), although there are some non-classical hydrogen bonding C—H···O interactions involving nitro and ether O atoms and H atoms from *sp*^2^ aromatic and vinylic C atoms, see Table 1. Unlike related compounds, the CH_2_ atoms do not act as hydrogen bond donors (Gerkin, 1999; Kennedy *et al.*, 2010). The asymmetric unit contains three independent molecules (Figure 2). Two have similar configurations but the third, that with C atoms numbered C16 through to C30, is somewhat more twisted. This is shown by the angle between the least squares planes of the aromatic rings (compare 70.26 (13) and 72.31 (12) with 84.22 (12) °). Related vinyl substituted benzyloxyphenyl species are known to have both twisted (Wang *et al.*, 2007; Zheng *et al.*, 2008) and planar conformations (Gao *et al.*, 2008; Stomberg & Lundquist, 1994) but in all cases, including the title complex, the vinyl group is coplanar with the attached aromatic ring. The three crystallographically independent molecules also differ from one another in the nature and number of their intermolecular contacts, Table 1. However, bond lengths and angles are in good agreement between the different configurations and with the related literature species.

### Experimental

All reactions in the preparation of 2-(4-benzyloxyphenyl)-1-nitroethene were performed under an atmosphere of nitrogen gas. 3.85 g, (18.16 mmol) of 4-benzyloxybenzaldehyde and 1.22 g of ammonium acetate (15.80 mmol) in nitromethane (100 ml) were heated at 343 K for 9 h. The solution was cooled and diluted with dichloromethane (54 ml), then washed with two 200 ml portions of saturated sodium chloride solution and then washed with of 200 ml distilled water. The resulting solution was dried over anhydrous magnesium sulfate and the solvent evaporated to dryness under reduced pressure. Recrystallization of the crude produce from ethanol gave yellow crystals (3.84 g, 83%), Mp: 387–388 K.

### Refinement

All H atoms were placed in calculated positions and refined in riding modes with *U*_iso_(H) = 1.2*U*_eq_(C). C—H distances 0.95 and 0.99 Å for CH and CH_2_ respectively.

### Crystal data

**Table:**

C_15_H_13_NO_3_	*Z* = 6
*M**_r_* = 255.26	*F*(000) = 804
Triclinic, *P*1	*D*_x_ = 1.345 Mg m^−^^3^
*a* = 9.9522 (8) Å	Mo *K*α radiation, λ = 0.71073 Å
*b* = 14.0456 (13) Å	Cell parameters from 78718 reflections
*c* = 14.2506 (10) Å	θ = 2.9–27.5°
α = 74.416 (5)°	µ = 0.09 mm^−^^1^
β = 84.188 (5)°	*T* = 120 K
γ = 80.888 (4)°	Cut slab, pale yellow
*V* = 1891.0 (3) Å^3^	0.12 × 0.10 × 0.05 mm

### Data collection

**Table:**

Bruker–Nonius APEXII CCD diffractometer	4735 reflections with *I* > 2σ(*I*)
Radiation source: Bruker-Nonius FR591 rotating anode	*R*_int_ = 0.063
10cm confocal mirrors	θ_max_ = 26.0°, θ_min_ = 3.0°
Detector resolution: 4096x4096pixels / 62x62mm pixels mm^-1^	*h* = −12→12
φ & ω scans	*k* = −17→17
25754 measured reflections	*l* = −17→17
7366 independent reflections	

### Refinement

**Table:**

Refinement on *F*^2^	Primary atom site location: structure-invariant direct methods
Least-squares matrix: full	Secondary atom site location: difference Fourier map
*R*[*F*^2^ > 2σ(*F*^2^)] = 0.088	Hydrogen site location: inferred from neighbouring sites
*wR*(*F*^2^) = 0.170	H-atom parameters constrained
*S* = 1.14	*w* = 1/[σ^2^(*F*_o_^2^) + (0.0086*P*)^2^ + 3.8491*P*] where *P* = (*F*_o_^2^ + 2*F*_c_^2^)/3
7366 reflections	(Δ/σ)_max_ < 0.001
514 parameters	Δρ_max_ = 0.27 e Å^−^^3^
0 restraints	Δρ_min_ = −0.28 e Å^−^^3^

### Special details

**Table:**

Geometry. All e.s.d.'s (except the e.s.d. in the dihedral angle between two l.s. planes) are estimated using the full covariance matrix. The cell e.s.d.'s are taken into account individually in the estimation of e.s.d.'s in distances, angles and torsion angles; correlations between e.s.d.'s in cell parameters are only used when they are defined by crystal symmetry. An approximate (isotropic) treatment of cell e.s.d.'s is used for estimating e.s.d.'s involving l.s. planes.
Refinement. Refinement of *F*^2^ against ALL reflections. The weighted *R*-factor *wR* and goodness of fit *S* are based on *F*^2^, conventional *R*-factors *R* are based on *F*, with *F* set to zero for negative *F*^2^. The threshold expression of *F*^2^ > σ(*F*^2^) is used only for calculating *R*-factors(gt) *etc*. and is not relevant to the choice of reflections for refinement. *R*-factors based on *F*^2^ are statistically about twice as large as those based on *F*, and *R*- factors based on ALL data will be even larger.

### Fractional atomic coordinates and isotropic or equivalent isotropic displacement parameters (Å^2^)

**Table:**

	*x*	*y*	*z*	*U*_iso_*/*U*_eq_	
O1	0.9330 (2)	0.37883 (19)	0.37919 (17)	0.0306 (6)	
O2	1.0417 (3)	−0.0863 (2)	0.9274 (2)	0.0468 (8)	
O3	0.8252 (3)	−0.0489 (2)	0.9037 (2)	0.0426 (7)	
O4	0.7595 (2)	0.6336 (2)	0.93137 (18)	0.0337 (6)	
O5	0.8521 (3)	1.0608 (2)	0.40189 (19)	0.0382 (7)	
O6	0.6349 (3)	1.1069 (2)	0.3947 (2)	0.0458 (8)	
O7	0.4798 (2)	0.3091 (2)	0.71568 (17)	0.0328 (6)	
O8	0.6922 (3)	−0.0535 (2)	1.2632 (2)	0.0462 (8)	
O9	0.4823 (3)	−0.0710 (2)	1.31177 (19)	0.0412 (7)	
N1	0.9451 (3)	−0.0400 (2)	0.8780 (2)	0.0342 (8)	
N2	0.7348 (3)	1.0530 (2)	0.4349 (2)	0.0320 (7)	
N3	0.5696 (3)	−0.0360 (2)	1.2495 (2)	0.0322 (8)	
C1	0.9316 (4)	0.3020 (3)	0.4612 (2)	0.0274 (8)	
C2	1.0458 (3)	0.2566 (3)	0.5139 (3)	0.0271 (8)	
H2	1.1326	0.2769	0.4915	0.033*	
C3	1.0318 (4)	0.1819 (3)	0.5989 (3)	0.0285 (8)	
H3	1.1099	0.1507	0.6343	0.034*	
C4	0.9047 (4)	0.1516 (3)	0.6337 (3)	0.0271 (8)	
C5	0.7925 (4)	0.1967 (3)	0.5792 (3)	0.0309 (9)	
H5	0.7056	0.1766	0.6014	0.037*	
C6	0.8055 (4)	0.2702 (3)	0.4936 (3)	0.0292 (8)	
H6	0.7282	0.2992	0.4567	0.035*	
C7	0.8843 (4)	0.0774 (3)	0.7258 (3)	0.0279 (8)	
H7	0.7942	0.0619	0.7438	0.034*	
C8	0.9796 (4)	0.0295 (3)	0.7866 (3)	0.0319 (9)	
H8	1.0716	0.0408	0.7700	0.038*	
C9	1.0559 (4)	0.4252 (3)	0.3534 (3)	0.0299 (9)	
H9A	1.1305	0.3788	0.3316	0.036*	
H9B	1.0846	0.4423	0.4107	0.036*	
C10	1.0263 (3)	0.5182 (3)	0.2724 (3)	0.0276 (8)	
C11	0.9907 (4)	0.5121 (3)	0.1823 (3)	0.0322 (9)	
H11	0.9836	0.4490	0.1723	0.039*	
C12	0.9657 (4)	0.5980 (3)	0.1072 (3)	0.0358 (10)	
H12	0.9412	0.5929	0.0462	0.043*	
C13	0.9756 (4)	0.6894 (3)	0.1196 (3)	0.0349 (9)	
H13	0.9580	0.7477	0.0678	0.042*	
C14	1.0117 (4)	0.6963 (3)	0.2085 (3)	0.0377 (10)	
H14	1.0200	0.7597	0.2176	0.045*	
C15	1.0359 (4)	0.6109 (3)	0.2849 (3)	0.0340 (9)	
H15	1.0591	0.6165	0.3460	0.041*	
C16	0.7608 (4)	0.7089 (3)	0.8479 (2)	0.0265 (8)	
C17	0.6479 (4)	0.7507 (3)	0.7931 (3)	0.0305 (9)	
H17	0.5614	0.7295	0.8147	0.037*	
C18	0.6632 (4)	0.8231 (3)	0.7073 (3)	0.0306 (9)	
H18	0.5859	0.8514	0.6702	0.037*	
C19	0.7888 (4)	0.8564 (3)	0.6729 (3)	0.0277 (8)	
C20	0.8995 (4)	0.8146 (3)	0.7303 (3)	0.0284 (8)	
H20	0.9861	0.8358	0.7092	0.034*	
C21	0.8855 (4)	0.7429 (3)	0.8170 (3)	0.0291 (9)	
H21	0.9617	0.7167	0.8558	0.035*	
C22	0.8072 (4)	0.9304 (3)	0.5808 (3)	0.0279 (8)	
H22	0.8975	0.9437	0.5594	0.033*	
C23	0.7081 (4)	0.9805 (3)	0.5246 (3)	0.0330 (9)	
H23	0.6170	0.9683	0.5441	0.040*	
C24	0.6366 (4)	0.5879 (3)	0.9591 (3)	0.0381 (10)	
H24A	0.6056	0.5710	0.9024	0.046*	
H24B	0.5634	0.6345	0.9819	0.046*	
C25	0.6680 (3)	0.4951 (3)	1.0395 (3)	0.0287 (9)	
C26	0.7175 (4)	0.4054 (3)	1.0175 (3)	0.0364 (10)	
H26	0.7309	0.4024	0.9514	0.044*	
C27	0.7477 (4)	0.3194 (3)	1.0922 (3)	0.0418 (10)	
H27	0.7823	0.2581	1.0766	0.050*	
C28	0.7279 (4)	0.3225 (3)	1.1887 (3)	0.0402 (10)	
H28	0.7468	0.2635	1.2395	0.048*	
C29	0.6802 (4)	0.4122 (3)	1.2100 (3)	0.0392 (10)	
H29	0.6683	0.4155	1.2761	0.047*	
C30	0.6495 (4)	0.4976 (3)	1.1359 (3)	0.0339 (9)	
H30	0.6151	0.5588	1.1518	0.041*	
C31	0.5003 (4)	0.2542 (3)	0.8087 (2)	0.0272 (8)	
C32	0.3980 (4)	0.2181 (3)	0.8771 (3)	0.0306 (9)	
H32	0.3055	0.2335	0.8608	0.037*	
C33	0.4313 (4)	0.1599 (3)	0.9687 (3)	0.0312 (9)	
H33	0.3611	0.1355	1.0150	0.037*	
C34	0.5662 (4)	0.1366 (3)	0.9941 (3)	0.0293 (8)	
C35	0.6680 (4)	0.1754 (3)	0.9255 (3)	0.0326 (9)	
H35	0.7604	0.1613	0.9421	0.039*	
C36	0.6354 (4)	0.2333 (3)	0.8347 (3)	0.0330 (9)	
H36	0.7053	0.2595	0.7892	0.040*	
C37	0.6052 (4)	0.0718 (3)	1.0889 (3)	0.0319 (9)	
H37	0.6991	0.0605	1.1013	0.038*	
C38	0.5207 (4)	0.0273 (3)	1.1591 (3)	0.0317 (9)	
H38	0.4259	0.0375	1.1494	0.038*	
C39	0.3446 (4)	0.3208 (3)	0.6810 (3)	0.0317 (9)	
H39A	0.2796	0.3651	0.7130	0.038*	
H39B	0.3113	0.2552	0.6951	0.038*	
C40	0.3582 (3)	0.3660 (3)	0.5727 (3)	0.0298 (9)	
C41	0.4149 (4)	0.3069 (3)	0.5108 (3)	0.0341 (9)	
H41	0.4414	0.2374	0.5365	0.041*	
C42	0.4326 (4)	0.3493 (3)	0.4114 (3)	0.0365 (10)	
H42	0.4705	0.3086	0.3691	0.044*	
C43	0.3955 (4)	0.4507 (3)	0.3735 (3)	0.0375 (10)	
H43	0.4090	0.4797	0.3055	0.045*	
C44	0.3390 (4)	0.5096 (3)	0.4347 (3)	0.0354 (9)	
H44	0.3132	0.5791	0.4089	0.043*	
C45	0.3199 (4)	0.4670 (3)	0.5338 (3)	0.0319 (9)	
H45	0.2801	0.5076	0.5757	0.038*	

### Atomic displacement parameters (Å^2^)

**Table:**

	*U*^11^	*U*^22^	*U*^33^	*U*^12^	*U*^13^	*U*^23^
O1	0.0257 (13)	0.0339 (16)	0.0294 (14)	−0.0089 (11)	−0.0034 (11)	0.0002 (12)
O2	0.0388 (17)	0.0464 (19)	0.0431 (17)	−0.0049 (14)	−0.0069 (14)	0.0104 (14)
O3	0.0341 (16)	0.0445 (18)	0.0417 (17)	−0.0109 (13)	0.0066 (13)	0.0016 (14)
O4	0.0312 (14)	0.0374 (16)	0.0290 (14)	−0.0146 (12)	−0.0044 (11)	0.0044 (12)
O5	0.0268 (14)	0.0438 (18)	0.0378 (16)	−0.0080 (12)	0.0017 (12)	0.0005 (13)
O6	0.0315 (16)	0.0483 (19)	0.0454 (17)	0.0029 (14)	−0.0119 (13)	0.0071 (14)
O7	0.0235 (13)	0.0436 (17)	0.0276 (14)	−0.0091 (12)	−0.0047 (10)	0.0006 (12)
O8	0.0308 (16)	0.059 (2)	0.0412 (17)	−0.0091 (14)	−0.0093 (13)	0.0045 (15)
O9	0.0378 (16)	0.0431 (18)	0.0361 (16)	−0.0109 (13)	0.0046 (13)	0.0017 (13)
N1	0.0334 (19)	0.0282 (19)	0.0377 (19)	−0.0061 (15)	0.0011 (15)	−0.0028 (15)
N2	0.0301 (18)	0.034 (2)	0.0288 (17)	−0.0060 (15)	−0.0038 (14)	−0.0022 (15)
N3	0.038 (2)	0.0303 (19)	0.0262 (17)	−0.0060 (15)	−0.0031 (14)	−0.0018 (14)
C1	0.030 (2)	0.027 (2)	0.0239 (19)	−0.0026 (16)	−0.0026 (15)	−0.0059 (16)
C2	0.0233 (18)	0.031 (2)	0.029 (2)	−0.0083 (16)	0.0009 (15)	−0.0090 (17)
C3	0.0261 (19)	0.029 (2)	0.028 (2)	−0.0006 (16)	−0.0049 (15)	−0.0036 (17)
C4	0.029 (2)	0.026 (2)	0.0272 (19)	−0.0054 (16)	−0.0015 (15)	−0.0071 (16)
C5	0.0248 (19)	0.032 (2)	0.036 (2)	−0.0105 (17)	0.0019 (16)	−0.0062 (18)
C6	0.0233 (19)	0.034 (2)	0.030 (2)	−0.0074 (16)	−0.0011 (15)	−0.0058 (17)
C7	0.0268 (19)	0.028 (2)	0.030 (2)	−0.0069 (16)	0.0003 (16)	−0.0076 (17)
C8	0.032 (2)	0.028 (2)	0.032 (2)	−0.0109 (17)	0.0018 (17)	0.0002 (17)
C9	0.0268 (19)	0.033 (2)	0.028 (2)	−0.0094 (17)	−0.0036 (15)	0.0000 (17)
C10	0.0179 (17)	0.032 (2)	0.033 (2)	−0.0075 (15)	−0.0007 (15)	−0.0066 (17)
C11	0.032 (2)	0.030 (2)	0.036 (2)	−0.0085 (17)	−0.0025 (17)	−0.0089 (18)
C12	0.033 (2)	0.046 (3)	0.027 (2)	−0.0087 (19)	−0.0079 (16)	−0.0023 (19)
C13	0.027 (2)	0.037 (3)	0.035 (2)	−0.0066 (18)	0.0005 (16)	0.0012 (19)
C14	0.037 (2)	0.026 (2)	0.048 (3)	−0.0057 (18)	−0.0028 (19)	−0.0046 (19)
C15	0.034 (2)	0.038 (3)	0.032 (2)	−0.0079 (18)	−0.0061 (17)	−0.0081 (18)
C16	0.0287 (19)	0.027 (2)	0.0235 (19)	−0.0093 (16)	−0.0039 (15)	−0.0026 (16)
C17	0.027 (2)	0.034 (2)	0.031 (2)	−0.0112 (17)	−0.0017 (16)	−0.0056 (18)
C18	0.0242 (19)	0.035 (2)	0.031 (2)	−0.0030 (17)	−0.0064 (15)	−0.0043 (18)
C19	0.030 (2)	0.024 (2)	0.028 (2)	−0.0050 (16)	−0.0020 (16)	−0.0051 (16)
C20	0.0242 (19)	0.029 (2)	0.031 (2)	−0.0067 (16)	−0.0023 (15)	−0.0031 (17)
C21	0.0259 (19)	0.032 (2)	0.029 (2)	−0.0050 (16)	−0.0050 (15)	−0.0049 (17)
C22	0.0256 (19)	0.030 (2)	0.028 (2)	−0.0066 (16)	0.0027 (15)	−0.0071 (17)
C23	0.030 (2)	0.037 (2)	0.030 (2)	−0.0100 (18)	0.0024 (16)	−0.0046 (18)
C24	0.032 (2)	0.044 (3)	0.036 (2)	−0.0197 (19)	−0.0011 (17)	0.0021 (19)
C25	0.0201 (18)	0.034 (2)	0.031 (2)	−0.0105 (16)	−0.0023 (15)	−0.0025 (17)
C26	0.028 (2)	0.047 (3)	0.034 (2)	−0.0096 (19)	0.0008 (17)	−0.010 (2)
C27	0.036 (2)	0.036 (3)	0.056 (3)	−0.0060 (19)	−0.003 (2)	−0.015 (2)
C28	0.031 (2)	0.038 (3)	0.043 (2)	−0.0072 (19)	−0.0041 (18)	0.006 (2)
C29	0.035 (2)	0.050 (3)	0.031 (2)	−0.014 (2)	0.0031 (17)	−0.006 (2)
C30	0.031 (2)	0.035 (2)	0.037 (2)	−0.0067 (17)	−0.0020 (17)	−0.0096 (19)
C31	0.032 (2)	0.025 (2)	0.0238 (19)	−0.0052 (16)	−0.0023 (15)	−0.0042 (16)
C32	0.0274 (19)	0.031 (2)	0.031 (2)	−0.0023 (16)	−0.0003 (16)	−0.0062 (17)
C33	0.031 (2)	0.036 (2)	0.026 (2)	−0.0092 (17)	0.0047 (16)	−0.0075 (18)
C34	0.033 (2)	0.026 (2)	0.028 (2)	−0.0063 (17)	−0.0012 (16)	−0.0052 (16)
C35	0.026 (2)	0.041 (2)	0.032 (2)	−0.0053 (17)	−0.0038 (16)	−0.0104 (18)
C36	0.029 (2)	0.042 (2)	0.026 (2)	−0.0076 (18)	0.0017 (16)	−0.0042 (18)
C37	0.030 (2)	0.032 (2)	0.034 (2)	−0.0033 (17)	0.0007 (17)	−0.0116 (18)
C38	0.030 (2)	0.034 (2)	0.029 (2)	−0.0006 (17)	−0.0053 (16)	−0.0069 (18)
C39	0.0252 (19)	0.038 (2)	0.029 (2)	−0.0053 (17)	−0.0062 (15)	−0.0024 (18)
C40	0.0206 (18)	0.036 (2)	0.032 (2)	−0.0080 (16)	−0.0051 (15)	−0.0042 (18)
C41	0.028 (2)	0.034 (2)	0.040 (2)	−0.0067 (17)	−0.0052 (17)	−0.0063 (19)
C42	0.028 (2)	0.045 (3)	0.040 (2)	−0.0072 (19)	−0.0011 (17)	−0.016 (2)
C43	0.030 (2)	0.050 (3)	0.029 (2)	−0.0078 (19)	−0.0063 (17)	−0.003 (2)
C44	0.028 (2)	0.038 (2)	0.035 (2)	−0.0027 (18)	−0.0040 (17)	0.0009 (19)
C45	0.0229 (19)	0.037 (2)	0.035 (2)	−0.0022 (17)	−0.0033 (16)	−0.0074 (18)

### Geometric parameters (Å, °)

**Table:**

O1—C1	1.362 (4)	C19—C22	1.454 (5)
O1—C9	1.445 (4)	C20—C21	1.379 (5)
O2—N1	1.239 (4)	C20—H20	0.9500
O3—N1	1.228 (4)	C21—H21	0.9500
O4—C16	1.364 (4)	C22—C23	1.321 (5)
O4—C24	1.441 (4)	C22—H22	0.9500
O5—N2	1.224 (4)	C23—H23	0.9500
O6—N2	1.239 (4)	C24—C25	1.500 (5)
O7—C31	1.359 (4)	C24—H24A	0.9900
O7—C39	1.451 (4)	C24—H24B	0.9900
O8—N3	1.231 (4)	C25—C30	1.376 (5)
O9—N3	1.237 (4)	C25—C26	1.383 (5)
N1—C8	1.449 (5)	C26—C27	1.393 (6)
N2—C23	1.435 (5)	C26—H26	0.9500
N3—C38	1.435 (5)	C27—C28	1.380 (6)
C1—C6	1.391 (5)	C27—H27	0.9500
C1—C2	1.396 (5)	C28—C29	1.376 (6)
C2—C3	1.385 (5)	C28—H28	0.9500
C2—H2	0.9500	C29—C30	1.385 (6)
C3—C4	1.399 (5)	C29—H29	0.9500
C3—H3	0.9500	C30—H30	0.9500
C4—C5	1.392 (5)	C31—C32	1.393 (5)
C4—C7	1.458 (5)	C31—C36	1.397 (5)
C5—C6	1.381 (5)	C32—C33	1.383 (5)
C5—H5	0.9500	C32—H32	0.9500
C6—H6	0.9500	C33—C34	1.392 (5)
C7—C8	1.324 (5)	C33—H33	0.9500
C7—H7	0.9500	C34—C35	1.402 (5)
C8—H8	0.9500	C34—C37	1.462 (5)
C9—C10	1.504 (5)	C35—C36	1.369 (5)
C9—H9A	0.9900	C35—H35	0.9500
C9—H9B	0.9900	C36—H36	0.9500
C10—C15	1.379 (5)	C37—C38	1.325 (5)
C10—C11	1.393 (5)	C37—H37	0.9500
C11—C12	1.388 (5)	C38—H38	0.9500
C11—H11	0.9500	C39—C40	1.504 (5)
C12—C13	1.362 (6)	C39—H39A	0.9900
C12—H12	0.9500	C39—H39B	0.9900
C13—C14	1.383 (5)	C40—C45	1.384 (5)
C13—H13	0.9500	C40—C41	1.389 (5)
C14—C15	1.393 (5)	C41—C42	1.386 (5)
C14—H14	0.9500	C41—H41	0.9500
C15—H15	0.9500	C42—C43	1.385 (6)
C16—C21	1.388 (5)	C42—H42	0.9500
C16—C17	1.392 (5)	C43—C44	1.378 (6)
C17—C18	1.377 (5)	C43—H43	0.9500
C17—H17	0.9500	C44—C45	1.385 (5)
C18—C19	1.400 (5)	C44—H44	0.9500
C18—H18	0.9500	C45—H45	0.9500
C19—C20	1.395 (5)		
			
C1—O1—C9	117.4 (3)	C23—C22—C19	125.1 (3)
C16—O4—C24	117.4 (3)	C23—C22—H22	117.4
C31—O7—C39	117.7 (3)	C19—C22—H22	117.4
O3—N1—O2	123.5 (3)	C22—C23—N2	121.6 (3)
O3—N1—C8	120.0 (3)	C22—C23—H23	119.2
O2—N1—C8	116.5 (3)	N2—C23—H23	119.2
O5—N2—O6	122.7 (3)	O4—C24—C25	107.8 (3)
O5—N2—C23	120.3 (3)	O4—C24—H24A	110.1
O6—N2—C23	117.0 (3)	C25—C24—H24A	110.1
O8—N3—O9	122.5 (3)	O4—C24—H24B	110.1
O8—N3—C38	121.0 (3)	C25—C24—H24B	110.1
O9—N3—C38	116.4 (3)	H24A—C24—H24B	108.5
O1—C1—C6	116.0 (3)	C30—C25—C26	118.9 (4)
O1—C1—C2	124.3 (3)	C30—C25—C24	121.0 (4)
C6—C1—C2	119.6 (3)	C26—C25—C24	120.2 (4)
C3—C2—C1	119.6 (3)	C25—C26—C27	120.2 (4)
C3—C2—H2	120.2	C25—C26—H26	119.9
C1—C2—H2	120.2	C27—C26—H26	119.9
C2—C3—C4	121.1 (3)	C28—C27—C26	120.6 (4)
C2—C3—H3	119.4	C28—C27—H27	119.7
C4—C3—H3	119.4	C26—C27—H27	119.7
C5—C4—C3	118.3 (3)	C29—C28—C27	119.0 (4)
C5—C4—C7	118.9 (3)	C29—C28—H28	120.5
C3—C4—C7	122.8 (3)	C27—C28—H28	120.5
C6—C5—C4	121.1 (3)	C28—C29—C30	120.5 (4)
C6—C5—H5	119.5	C28—C29—H29	119.8
C4—C5—H5	119.5	C30—C29—H29	119.8
C5—C6—C1	120.2 (3)	C25—C30—C29	120.9 (4)
C5—C6—H6	119.9	C25—C30—H30	119.5
C1—C6—H6	119.9	C29—C30—H30	119.5
C8—C7—C4	126.1 (3)	O7—C31—C32	124.9 (3)
C8—C7—H7	116.9	O7—C31—C36	115.8 (3)
C4—C7—H7	116.9	C32—C31—C36	119.3 (3)
C7—C8—N1	120.8 (3)	C33—C32—C31	120.0 (3)
C7—C8—H8	119.6	C33—C32—H32	120.0
N1—C8—H8	119.6	C31—C32—H32	120.0
O1—C9—C10	108.1 (3)	C32—C33—C34	120.9 (3)
O1—C9—H9A	110.1	C32—C33—H33	119.5
C10—C9—H9A	110.1	C34—C33—H33	119.5
O1—C9—H9B	110.1	C33—C34—C35	118.6 (3)
C10—C9—H9B	110.1	C33—C34—C37	122.2 (3)
H9A—C9—H9B	108.4	C35—C34—C37	119.2 (3)
C15—C10—C11	118.6 (3)	C36—C35—C34	120.7 (4)
C15—C10—C9	121.0 (3)	C36—C35—H35	119.7
C11—C10—C9	120.4 (3)	C34—C35—H35	119.7
C12—C11—C10	120.3 (4)	C35—C36—C31	120.5 (3)
C12—C11—H11	119.9	C35—C36—H36	119.7
C10—C11—H11	119.9	C31—C36—H36	119.7
C13—C12—C11	121.1 (4)	C38—C37—C34	125.7 (4)
C13—C12—H12	119.5	C38—C37—H37	117.2
C11—C12—H12	119.5	C34—C37—H37	117.2
C12—C13—C14	119.2 (4)	C37—C38—N3	121.3 (4)
C12—C13—H13	120.4	C37—C38—H38	119.3
C14—C13—H13	120.4	N3—C38—H38	119.3
C13—C14—C15	120.4 (4)	O7—C39—C40	106.1 (3)
C13—C14—H14	119.8	O7—C39—H39A	110.5
C15—C14—H14	119.8	C40—C39—H39A	110.5
C10—C15—C14	120.5 (4)	O7—C39—H39B	110.5
C10—C15—H15	119.8	C40—C39—H39B	110.5
C14—C15—H15	119.8	H39A—C39—H39B	108.7
O4—C16—C21	115.9 (3)	C45—C40—C41	119.2 (3)
O4—C16—C17	124.4 (3)	C45—C40—C39	120.8 (3)
C21—C16—C17	119.7 (3)	C41—C40—C39	120.0 (4)
C18—C17—C16	119.2 (3)	C42—C41—C40	120.0 (4)
C18—C17—H17	120.4	C42—C41—H41	120.0
C16—C17—H17	120.4	C40—C41—H41	120.0
C17—C18—C19	122.2 (3)	C43—C42—C41	120.3 (4)
C17—C18—H18	118.9	C43—C42—H42	119.9
C19—C18—H18	118.9	C41—C42—H42	119.9
C20—C19—C18	117.3 (3)	C44—C43—C42	119.9 (4)
C20—C19—C22	120.0 (3)	C44—C43—H43	120.0
C18—C19—C22	122.7 (3)	C42—C43—H43	120.0
C21—C20—C19	121.2 (3)	C43—C44—C45	119.8 (4)
C21—C20—H20	119.4	C43—C44—H44	120.1
C19—C20—H20	119.4	C45—C44—H44	120.1
C20—C21—C16	120.3 (3)	C40—C45—C44	120.8 (4)
C20—C21—H21	119.8	C40—C45—H45	119.6
C16—C21—H21	119.8	C44—C45—H45	119.6
			
C9—O1—C1—C6	169.3 (3)	C19—C22—C23—N2	179.5 (3)
C9—O1—C1—C2	−9.1 (5)	O5—N2—C23—C22	9.6 (6)
O1—C1—C2—C3	177.1 (3)	O6—N2—C23—C22	−169.3 (4)
C6—C1—C2—C3	−1.3 (5)	C16—O4—C24—C25	168.5 (3)
C1—C2—C3—C4	−0.7 (6)	O4—C24—C25—C30	91.2 (4)
C2—C3—C4—C5	1.6 (6)	O4—C24—C25—C26	−88.2 (4)
C2—C3—C4—C7	−176.3 (3)	C30—C25—C26—C27	0.0 (5)
C3—C4—C5—C6	−0.6 (6)	C24—C25—C26—C27	179.4 (3)
C7—C4—C5—C6	177.4 (3)	C25—C26—C27—C28	0.5 (6)
C4—C5—C6—C1	−1.3 (6)	C26—C27—C28—C29	−1.3 (6)
O1—C1—C6—C5	−176.2 (3)	C27—C28—C29—C30	1.6 (6)
C2—C1—C6—C5	2.3 (6)	C26—C25—C30—C29	0.3 (5)
C5—C4—C7—C8	−178.6 (4)	C24—C25—C30—C29	−179.1 (3)
C3—C4—C7—C8	−0.6 (6)	C28—C29—C30—C25	−1.1 (6)
C4—C7—C8—N1	177.6 (3)	C39—O7—C31—C32	−8.1 (5)
O3—N1—C8—C7	−6.0 (6)	C39—O7—C31—C36	171.5 (3)
O2—N1—C8—C7	175.5 (4)	O7—C31—C32—C33	177.7 (4)
C1—O1—C9—C10	−170.1 (3)	C36—C31—C32—C33	−1.8 (6)
O1—C9—C10—C15	118.0 (4)	C31—C32—C33—C34	0.1 (6)
O1—C9—C10—C11	−63.0 (4)	C32—C33—C34—C35	1.5 (6)
C15—C10—C11—C12	0.0 (5)	C32—C33—C34—C37	−177.4 (4)
C9—C10—C11—C12	−179.0 (3)	C33—C34—C35—C36	−1.2 (6)
C10—C11—C12—C13	0.2 (6)	C37—C34—C35—C36	177.6 (4)
C11—C12—C13—C14	0.2 (6)	C34—C35—C36—C31	−0.5 (6)
C12—C13—C14—C15	−0.8 (6)	O7—C31—C36—C35	−177.5 (3)
C11—C10—C15—C14	−0.7 (5)	C32—C31—C36—C35	2.0 (6)
C9—C10—C15—C14	178.3 (3)	C33—C34—C37—C38	1.3 (6)
C13—C14—C15—C10	1.0 (6)	C35—C34—C37—C38	−177.5 (4)
C24—O4—C16—C21	−172.4 (3)	C34—C37—C38—N3	179.3 (3)
C24—O4—C16—C17	6.4 (5)	O8—N3—C38—C37	−2.5 (6)
O4—C16—C17—C18	−176.7 (3)	O9—N3—C38—C37	177.4 (4)
C21—C16—C17—C18	2.1 (6)	C31—O7—C39—C40	−169.0 (3)
C16—C17—C18—C19	0.0 (6)	O7—C39—C40—C45	−100.5 (4)
C17—C18—C19—C20	−1.1 (6)	O7—C39—C40—C41	76.8 (4)
C17—C18—C19—C22	177.9 (4)	C45—C40—C41—C42	0.1 (5)
C18—C19—C20—C21	0.3 (6)	C39—C40—C41—C42	−177.3 (3)
C22—C19—C20—C21	−178.7 (3)	C40—C41—C42—C43	0.7 (5)
C19—C20—C21—C16	1.7 (6)	C41—C42—C43—C44	−0.8 (6)
O4—C16—C21—C20	176.0 (3)	C42—C43—C44—C45	0.2 (6)
C17—C16—C21—C20	−2.9 (6)	C41—C40—C45—C44	−0.8 (5)
C20—C19—C22—C23	−174.4 (4)	C39—C40—C45—C44	176.6 (3)
C18—C19—C22—C23	6.6 (6)	C43—C44—C45—C40	0.6 (6)

### Hydrogen-bond geometry (Å, °)

**Table:**

*D*—H···*A*	*D*—H	H···*A*	*D*···*A*	*D*—H···*A*
C3—H3···O8^i^	0.95	2.56	3.503 (5)	175
C8—H8···O8^i^	0.95	2.38	3.322 (5)	175
C5—H5···O9^ii^	0.95	2.58	3.493 (4)	162
C12—H12···O4^iii^	0.95	2.46	3.284 (5)	145
C20—H20···O5^iv^	0.95	2.49	3.373 (4)	155
C22—H22···O5^iv^	0.95	2.59	3.448 (4)	150
C18—H18···O6^v^	0.95	2.40	3.326 (4)	166
C33—H33···O3^ii^	0.95	2.40	3.325 (4)	163
C45—H45···O1^vi^	0.95	2.58	3.413 (5)	147

Symmetry codes: (i) −*x*+2, −*y*, −*z*+2; (ii) −*x*+1, −*y*, −*z*+2; (iii) *x*, *y*, *z*−1; (iv) −*x*+2, −*y*+2, −*z*+1; (v) −*x*+1, −*y*+2, −*z*+1; (vi) −*x*+1, −*y*+1, −*z*+1.
